# Direct Estimation of Choroidal Thickness in Optical Coherence Tomography Images with Convolutional Neural Networks

**DOI:** 10.3390/jcm11113203

**Published:** 2022-06-04

**Authors:** Yibiao Rong, Zehua Jiang, Weihang Wu, Qifeng Chen, Chuliang Wei, Zhun Fan, Haoyu Chen

**Affiliations:** 1 College of Engineering, Shantou University, Shantou 515063, China; ybrong@stu.edu.cn (Y.R.); 20whwu1@stu.edu.cn (W.W.); 16qfchen@stu.edu.cn (Q.C.); clwei@stu.edu.cn (C.W.); 2 Key Laboratory of Digital Signal and Image Processing of Guangdong Provincial, Shantou University, Shantou 515063, China; 3 Joint Shantou International Eye Center, Shantou University and the Chinese University of Hong Kong, Shantou 515051, China; jzh@jsiec.org; 4 Medical College, Shantou University, Shantou 515063, China

**Keywords:** choroidal thickness, optical coherence tomography, direct estimation, convolutional neural networks

## Abstract

Automatic and accurate estimation of choroidal thickness plays a very important role in a computer-aided system for eye diseases. One of the most common methods for automatic estimation of choroidal thickness is segmentation-based methods, in which the boundaries of the choroid are first detected from optical coherence tomography (OCT) images. The choroidal thickness is then computed based on the detected boundaries. A shortcoming in the segmentation-based methods is that the estimating precision greatly depends on the segmentation results. To avoid the dependence on the segmentation step, in this paper, we propose a direct method based on convolutional neural networks (CNNs) for estimating choroidal thickness without segmentation. Concretely, a B-scan image is first cropped into several patches. A trained CNN model is then used to estimate the choroidal thickness for each patch. The mean thickness of the choroid in the B-scan is obtained by taking the average of the choroidal thickness on each patch. Then, 150 OCT volumes are collected to evaluate the proposed method. The experiments show that the results obtained by the proposed method are very competitive with those obtained by segmentation-based methods, which indicates that direct estimation of choroidal thickness is very promising.

## 1. Introduction

The choroid, the posterior segment of the uveal tract, is a layer of blood vessels and connective tissue between the retina and sclera. It plays an important role in many physiologic processes, including nourishing the retina and prelaminar optic nerve, acting as a light absorber, and working as a heat sink [1,2]. The choroidal thickness changes with the development of the eyes and can be employed to assist in the diagnosis of retinal and choroidal disorders. Some diseases, such as central serous chorioretinopathy and Vogt–Koyanagi–Harada diseases, are related to an anomalous thick choroid. Some others, such as myopic maculopathy, are correlated with an abnormally thin choroid [3]. 

Precise visualization of the choroid can be achieved with an optical coherence tomography (OCT) machine with technologies that can increase penetration and decrease the backscatter of the retinal pigment epithelium, such as enhanced depth imaging OCT and swept-source OCT [4,5]. To measure the choroidal thickness accurately, once the OCT images are obtained, a trained doctor is generally required to delineate the boundaries of the choroid. However, manually delineating the boundaries of the choroid is an exhausting task.

To relieve the burden on the doctors, many automatic methods for estimating choroidal thickness have been developed, among which segmentation-based methods are the most common at present. In the segmentation-based methods, the boundaries of the choroid are first detected. The thickness is then computed based on the detected boundaries. In the process, designing choroidal segmentation methods is the key step. Before the revival of deep learning [6], especially deep convolutional neural networks [7], graph-cut/search-based methods [8] were popular for the segmentation of choroids. Hu et al. [9] employed a graph-based, multistage segmentation approach to identify the boundaries of the choroid. Tian et al. [10] presented an algorithm that could segment the choroid automatically based on graph search methods. Chen et al. [11] proposed an improved 2D graph-search method with curve smooth constraints to obtain the choroidal segmentation. Danesh et al. [12] designed a multiresolution, texture-based model with graph cuts for segmentation of choroidal boundaries.

With the development of deep learning, designing methods for choroidal segmentation based on deep neural networks, including fully convolutional networks [13], SegNet [14], and U-Net [15], has become mainstream. Mao et al. [16] designed a skip connection attention module and integrated it into the U-Net architecture to segment choroidal boundaries. Zhang et al. [17] proposed a biomarker-infused global-to-local network for choroidal segmentation. Cheng et al. [18] designed a refinement residual block and a channel attention block to improve the ability of U-Net on segmentation of choroid. Kugelman et al. [19] presented a variety of patch-based and fully convolutional deep learning methods to accurately determine the location of the choroidal boundaries. Moreover, some researchers have also tried to combine the deep learning techniques and graph cut/search for segmentation of choroid. For example, Sui et al. [20] presented a multiscale and end-to-end convolutional network architecture to learn an optimal graph-edge weight for detecting choroidal boundaries.

Notably, the choroidal thickness can only be computed after choroidal segmentation in segmentation-based methods. In the process of choroidal segmentation, several intermediate steps might also be included. For example, in the patch-based method presented in [19], the authors first employed CNNs to obtain the probability maps. Graph search was then applied to obtain the choroidal boundaries. As the choroidal thickness is computed based on the detected boundaries, the estimating precision in segmentation-based methods greatly depends on the intermediate steps. In other words, if an algorithm cannot detect the boundaries of a choroid correctly, the computed choroidal thickness based on the detected boundaries might not be correct either. In this paper, we propose a direct method without segmentation for estimating choroidal thickness based on CNNs. As the proposed method is segmentation-free, it can avoid being dependent on segmentation steps and instead directly focuses on the final clinical purpose.

Employing direct methods without segmentation to estimate object properties can be found in other medical imaging domains. For example, Wang et al. [21] proposed a method for direct estimation of cardiac biventricular volumes with an adapted Bayesian formulation. With the revival of deep learning, some researchers have also employed deep neural networks for the direct estimation of object properties from medical images. Zhen et al. [22] presented a multiscale deep network and regression forest for direct biventricular volume estimation. Luo et al. [23] proposed a new network structure for end-to-end left ventricular volume estimation on cardiac MR images. Manit et al. [24] presented a deep CNN approach for forehead tissue thickness estimation on laser backscattering images. Zhang et al. [25] proposed a CNN-based regression model to learn inherent associations between local image patches and target anatomical landmarks. Vos et al. [26] employed CNNs for direct coronary calcium scoring in cardiac and chest CT images. Mariottoni et al. [27] presented a deep learning segmentation-free method for quantification of retinal nerve fiber layer thickness on OCT images. 

However, to the best of our knowledge, there is little published research on the estimation of choroidal thickness in OCT images without segmentation. In this study, the characteristics of the choroid, e.g., its shape, are different from the objectives reported in existing studies [21,22,23,24,25,26,27]. In addition, the imaging technologies used to obtain the data needed for different objectives are also different. Thus, a question arises as to whether direct methods are also effective for estimating the choroidal thickness based on OCT images. To explore this issue, in this paper, we propose a method to estimate the choroidal thickness in OCT images directly based on CNNs, making this the first study to estimate the choroidal thickness in OCT images without segmentation. 

## 2. Materials and Methods

### 2.1. Patients

This study was approved by the Institutional Review Board (IRB) of Joint Shantou International Eye Center, Shantou University, and the Chinese University of Hong Kong. Due to the retrospective nature of the study, informed consent was waived by the IRB. 

We collected 150 OCT volumes from 150 eyes with Topcon Triton, a swept-source OCT. Each OCT volume consists of 256 B-scans with a size of 596 × 800 × 3, covering a 7 × 7 × 2.6 mm^3^ region centered on the fovea. The choroidal boundaries in each B-scan were automatically delineated using the inbuilt algorithm of the Topcon system and then revised by a trained doctor if the inbuilt algorithm could not detect the boundaries correctly. The ground truth of choroidal thickness was computed based on the delineated boundaries. To train and test the CNN models, the OCT volumes were randomly divided into a training set, validation set, and test set with a ratio of 60–20–20%. Specifically, 90 OCT volumes were selected as the training set, 30 OCT volumes, as the validation set, and 30 as the test set. It is worth noting that the training, validation, and test sets were organized randomly by subjects. Participants were different across different datasets. The numbers of B-scans in the training, validation, and test sets were 90 × 256 = 23,040, 30 × 256 = 7680, and 30 × 256 = 7680, respectively. In the training, validation, and test sets, the mean choroidal thicknesses were 57.8279 (pixels), 57.8356 (pixels), and 69.2251 (pixels), respectively, and the ranges were 18 to 109 (pixels), 19 to 109 (pixels), and 32 to 146 (pixels), respectively.

### 2.2. Design of Algorithm

**Figure 1:**
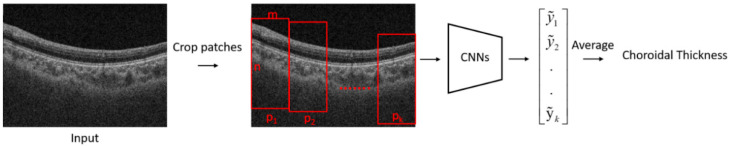
The flowchart of the proposed method for estimation of choroidal thickness. A B-scan image was first cropped into several patches. A trained CNN model was then used to estimate the choroidal thickness for each patch. The mean thickness of the choroid in the B-scan was obtained by taking the average of the choroidal thickness on each patch. m and n are the width and height of a patch p respectively.

The flowchart of the proposed algorithm for the estimation of choroidal thickness is shown in Figure 1. A B-scan image was first cropped into several patches. A trained CNN model was then used to estimate the choroidal thickness for each patch. The mean thickness of the choroid in the B-scan was obtained by taking the average of the choroidal thickness on each patch.

### 2.3. The Process of Cropping Images

A large part of a retinal OCT image is the background or regions of vitreous, which do not contain useful information for estimating the choroidal thickness. If we employ a whole B-scan as the input for a CNN model, it would lead to memory consumption. In addition, the noise from the background would deteriorate the performance of CNNs. Moreover, if we predict the mean value of an entire choroidal region in a B-scan image directly, the mean value may not be representative of the complex structure of the choroid. To handle the problems caused by using the whole B-scan as input, we propose using patches as the input for a CNN model. As a patch is cropped from a B-scan, the size of a patch is smaller, thus saving more memory. In addition, while cropping the patches, we could make a patch containing the foreground while excluding the background as much as possible. As a result, the influence of the noise from the background is restricted. Moreover, in a small regional range, the choroidal thickness would not change considerably; thus, employing a mean value to represent the choroidal thickness in a small regional range is reasonable to some extent.

**Figure 2:**
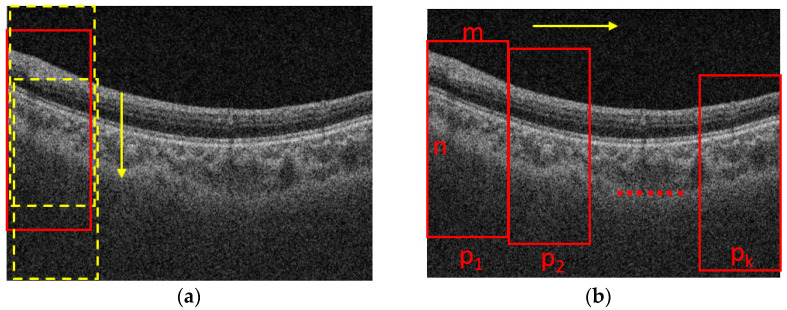
The process for cropping images. (**a**) sliding in the vertical direction; (**b**) sliding in the horizontal direction. m and n are the width and height of a patch p respectively.

The process for cropping the images is shown in Figure 2. Assume that the size of a B-scan image is N × M, where N is the height and M is the width of the B-scan image, respectively. The B-scan image is first divided into K segments in horizontal direction equally. The width of a patch is m = M/K. The height of a patch is n (n < N), which is set based on the rule that the patch should contain the foreground while excluding the background as much as possible. To determine the location of a patch in the vertical direction, we slide the window with a size of n × m from top to end in a B-scan image with a fixed stride, e.g., 10 pixels, as shown in Figure 2a, and the one with maximal summed intensity is selected as the final patch. Then, we slide the window into the next patch with the stride of m in the horizontal direction, as shown in Figure 2b, and repeat the process shown in Figure 2a. The process shown in Figure 2b is also repeated until the window reaches the rightmost side of the B-scan. It is worth noting that there is no intersection between two adjacent patches, so it is convenient for us to take the average choroidal thickness on each patch to compute the mean thickness of the choroid in the whole B-scan.

### 2.4. Network’s Details for Choroidal Thickness Estimation

Convolutional neural networks (CNNs) can be employed to solve different kinds of problems by constructing a proper network structure $R_{\theta }$ and cost function ϕ given the training set ${\left\{(x_{s},y_{s})\right\}}_{s=1}^{S}$, where $x_{s}$ is the observed value, and $y_{s}$ is the desired output [7]. This process can be formulated as

(1) $$R_{learn}=\underset{R_{\theta },\theta \in \Theta }{\min}(\sum_{s=1}^{S}\phi (R_{\theta }(x_{s}),y_{s}))$$

where Θ is the set of all possible parameters. Once the learning step is completed, $R_{learn}$ can then be used for a specific task, e.g., classification, regression, etc. In this study, we employed CNNs to predict choroidal thickness based on OCT images. Thus, $x_{s}$ and $y_{s}$ represent an image patch and corresponding choroidal thickness, respectively. Mean absolute error is used as the loss function for CNNs—namely, $\phi (R_{\theta }(x_{s}),y_{s})=|R_{\theta }(x_{s})-y_{s}|$.

**Figure 3:**
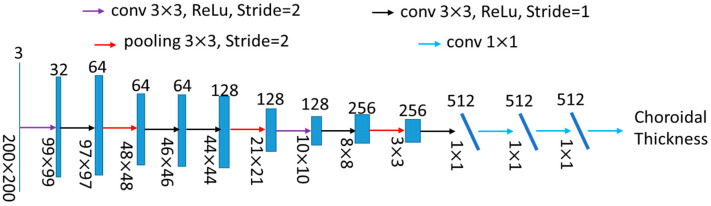
The network architecture applied in this work.

Particularly, the architecture of a typical CNN model includes several convolutional layers and optional pooling layers, followed by at least one fully connected layer. For the details about CNNs, we refer readers to the tutorial developed by Vedaldi et al. [28]. The CNN models applied in this study are shown in Figure 3. There were 7 convolutional layers with a kernel size of 3 × 3. Each convolutional layer was followed by a ReLu layer [29], which is not shown in Figure 3. Every two convolutional layers were followed by a pooling layer to downsample the feature maps. Finally, a fully connected layer was employed to map each component to the final prediction. It is worth noting that the CNN shown in Figure 3 is for the patches with a size of 200 × 200 × 3. For the patches with other sizes in the experimental section, the parameters, e.g., stride, would be changed while keeping the network architecture the same.

### 2.5. Training Details and Parameter Setting

A PC, equipped with an Intel (R) Xeon (R) W-223 CPU at 3.60 GHz, 32 GB of RAM capacity, and NVIDIA Quadro P2200, was employed to perform the experiments with MATLAB. We used MatConvNet [28] to train the CNNs. The parameters of CNNs were initialized with the Xavier initialization method and trained for 41 epochs with a mini-batch size of 15 instances. Training convergence can be observed within 31 epochs. For other hyperparameters, learning rates were set to 0.002 for the first 11 epochs, 0.0002 for the 12th to 27th epochs, and 0.00002 for the last few epochs. The momentum was 0.9, and the weight decay was 0.0005.

### 2.6. Metrics

The metrics used to evaluate the proposed method were mean absolute error (MAE), mean error (ME), and Poisson correlation coefficient (PCC) [30,31]. MAE and ME are quantities used to measure how close predictions are to the eventual outcomes, which are defined as

(2) $$MAE=\frac{1}{S}\sum_{s=1}^{S}\left|f_{s}-y_{s}\right|$$

(3) $$ME=\frac{1}{S}\sum_{s=1}^{S}(f_{s}-y_{s})$$

where $f_{s}$ is the estimated result obtained by the algorithm, and $y_{s}$ is the ground truth. A smaller MAE or ME indicates better performance. In this paper, MAE or ME represents the pixel-wise error between the predicted results and the ground truth. PCC is a measure of the linear correlation between two variables (predicted results and ground truth in this paper), defined as

(4) $$PCC=\frac{\sum_{s=1}^{S}\left(f_{s}-\bar{f}\right)(y_{s}-\bar{y})}{\sqrt{\sum_{s=1}^{S}(f_{s}-\bar{f}{)}^{2}{\left(y_{s}-\bar{y}\right)}^{2}}}$$

It has a value between +1 and −1, where 1 is a total positive linear correlation, 0 is no linear correlation, and −1 is a total negative linear correlation. A higher correlation coefficient indicates better performance.

## 3. Results

In the proposed method, a B-scan image was first cropped into several patches, and a trained CNN model was then employed to estimate the choroidal thickness for each patch. The mean thickness of the choroid in the B-scan was obtained by taking the average of the choroidal thickness on each patch. In this process, there were two types of choroidal thickness that needed to be estimated, one corresponding to the patch and another to the B-scan. Thus, we evaluated the performance of the proposed method at the patch and B-scan levels, respectively. 

**Figure 4:**
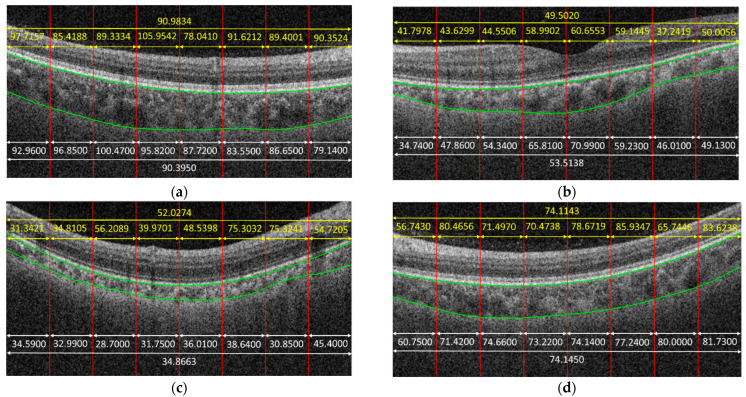

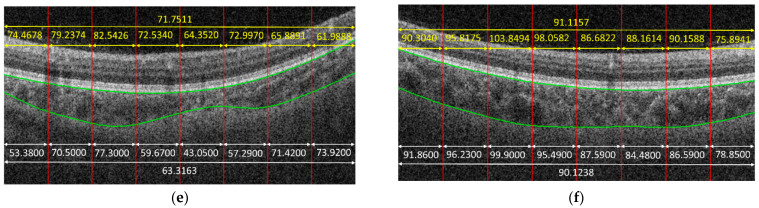
Examples to demonstrate the predicted results obtained by the proposed method, in which yellow parts are the results obtained by the proposed method. White parts are the ground truth. Green curves are the boundaries of choroid. (**a**) MAE = 0.5884, (**b**) MAE = 4.0118, (**c**) MAE = 17.1611, (**d**) MAE = 0.0307, (**e**) MAE = 8.4348, (**f**) MAE = 0.9919.

Figure 4 shows some examples to demonstrate the predicted results obtained by the proposed method. In the given examples, eight patches were cropped from a B-scan. The predicted result for each patch is shown in yellow color. The average on the predicted results of each patch cropped from the same B-scan is also in yellow. The white parts in Figure 4 are the corresponding ground truth, and green curves are the boundaries of the choroid. It is observed that the predicted results obtained by the proposed method were very close to the ground truth for some cases, e.g., Figure 4f, while the proposed method might also fail for some other cases, e.g., Figure 4c.

Table 1 summarizes the quantitative results obtained at the patch and B-scan levels, respectively. It is observed that different patch sizes would lead to different performances. It is worth noting that the size of a patch is represented as n × m, where n represents the vertical value, and m represents the horizontal value. When the patch size was 200 × 50, i.e., when 16 patches were cropped from a B-scan, the average MAE was 7.1197, with a standard deviation of 9.1715, and PCC was 0.8644 at the patch level. At the B-scan level, MAE was 4.5871, with a standard deviation of 6.4419, and PCC was 0.9193. When the patch size was increased to 200 × 200, i.e., when four patches were cropped from a B-scan, the performance improved, compared with the performance of the patch size of 200 × 50. However, this does not mean that the larger the patch size, the better. As can be seen in Table 1, although the MAE decreased at the patch level when the patch size was 400 × 400, the performance deteriorated, compared with the performance of patches with a size of 200 × 200 at the B-scan level. In addition, the larger the patch size, the less local information could be obtained. Thus, 200 × 200 was a good choice that could guarantee the proposed method achieves high accuracy but also could ensure the proposed method provides relatively much more local information. 

From Table 1, we can also observe that the ME value was negative for patches with sizes of 200 × 50, 200 × 100, and 200 × 200, which indicates that the CNNs tended to underestimate the values of choroidal thickness for these cases. While for patches with a size of 400 × 400, the ME value was positive, which means that the CNNs tended to overestimate the values. Another phenomenon observed in Table 1 is that the performance at the B-scan level was always better than the performance at the patch level regardless of the patch size. The reason is that the mean thickness of choroid in a B-scan is computed by taking the average of the thicknesses on the patches cropped from that B-scan, which makes the errors in patch level offset each other to some extent. Figure 4d gives an example; as can be observed, the trained CNN model could not predict the choroidal thickness correctly for some patches—namely, the predicted results were larger or smaller, compared with the ground truth. However, when we took the average, the biases in larger and smaller cases offset each other; thus, the precision of choroidal thickness at the B-scan level improved, compared with that at the patch level.

**Figure 5:**
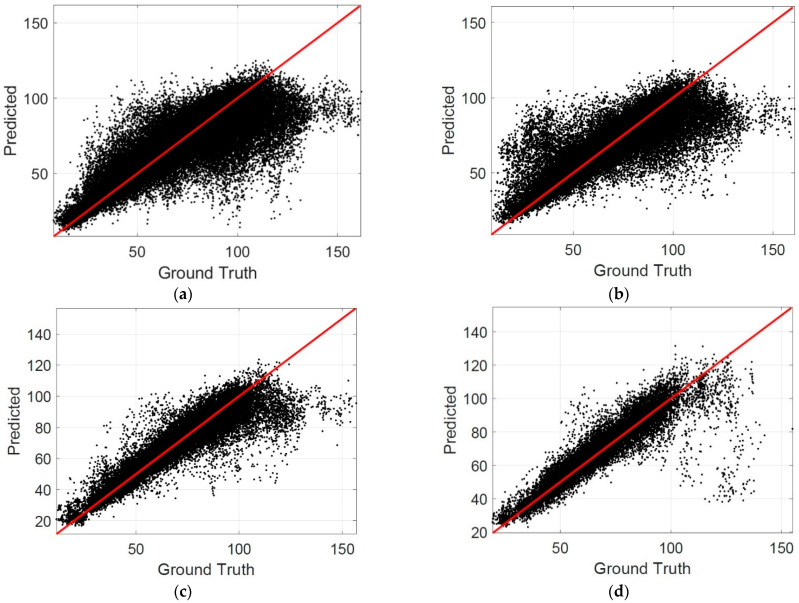
The scatterplots in patch level: (**a**) the patch size is 200 × 50; (**b**) 200 × 100; (**c**) 200 × 200; (**d**) 400 × 400.

The scatterplots are also drawn to evaluate the performance of the proposed method. In a scatterplot, the difference between the predicted results and the ground truth can be observed clearly. The more points close to the identical line in a scatterplot indicate a better performance of the proposed method. Figure 5 shows the scatterplots for the patch level, where the horizontal axis represents the ground truth, and the vertical axis represents the predicted results. Table 2 summarizes the percentage of patches in different MAE intervals. It is observed that the percentage of patches in the interval of MAE ≤ 5 reached 0.6153 when the patch size was 200 × 200, which is the best among the listed cases. The percentage of patches in the interval of MAE ≤ 10 was larger than 0.8 when the patch size was 200 × 200, which indicates that the choroidal thickness can be estimated correctly by the CNN models for most patches.

**Figure 6:**
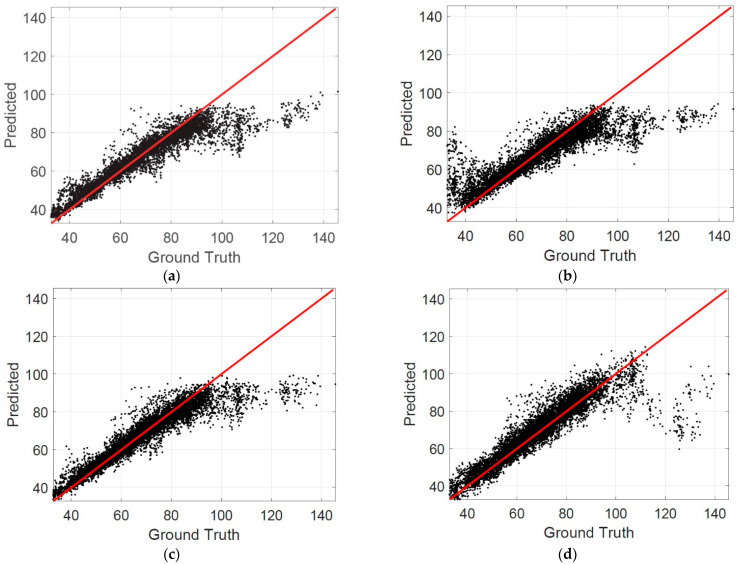
The scatterplots in B-scan level: (**a**) rhe patch size is 200 × 50; (**b**) 200 × 100; (**c**) 200 × 200; (**d**) 400 × 400.

Figure 6 shows the scatterplots at the B-scan level. Table 3 summarizes the percentage of B-scans in different MAE intervals. It is observed that the percentage of B-scan in the interval of MAE ≤ 10 was 0.9086 when the patch size was 200 × 200, which also indicates that the proposed method can estimate the choroidal thickness correctly for most B-scans.

## 4. Discussion

In this paper, we proposed a direct method to estimate the choroidal thickness in OCT images based on CNNs. In the proposed method, a B-scan image was first cropped into several patches, which were taken as the input for a trained CNN model. The output of the CNN model was the mean thickness of the choroid in a patch. The mean thickness of choroid in a B-scan was obtained by computing the average of the choroidal thickness on the patches cropped from that B-scan. Notably, there is no segmentation step in the proposed method, which makes the proposed method independent of the segmentation step and puts focus on the final clinical purpose. We collected 38400 B-scan images to evaluate the proposed direct method. The experiments show that the predicted values were very close to the ground truth when MAE ≤ 10, and the points were almost located in the identical line when MAE ≤ 5, which indicates that a CNN model for choroidal thickness estimation would be acceptable in clinical practice if it achieves the performance with MAE ≤ 10. The percentages of B-scans in the intervals with MAE ≤ 5 and MAE ≤ 10 achieved by the proposed method were 0.7530 and 0.9086, respectively, which means that the proposed method can estimate the choroidal thickness correctly in a direct way for most B-scans.

To evaluate the performance of the proposed direct method further, several segmentation-based methods for choroidal thickness estimation, including deep-learning-based methods and graph-cut-based methods [12,15,16,17], were selected for comparison. Notably, in segmentation-based methods, there are two boundaries—the Bruch’s membrane (BM) and choroid–sclera interface (CSI) boundaries—to be detected to compute the choroidal thickness. Table 4 summarizes the results, where BM and CSI represent the pixel-wise mismatch between the detected results and the ground truth in the segmentation-based methods. MAE represents the pixel-wise error of choroidal thickness between the results obtained by different methods and ground truth at the B-scan level. The best results reported in the corresponding literature are cited directly for comparison. If a metric was not reported in the corresponding literature, it appears as a gap in Table 4. It is observed that the results obtained by the proposed direct method are very competitive with those obtained by segmentation-based methods, including the Bio-Net [17], which indicates that the direct estimation of the choroidal thickness without segmentation is very promising.

Although the results obtained by the proposed direct method are very competitive, because of the black-box nature of the CNNs, it is difficult to judge in which cases the proposed method can estimate the thickness correctly and in which it cannot, which is a major limitation for the proposed direct method, compared with segmentation-based methods. In segmentation-based methods, the detected choroidal boundaries can be visualized; thus, a trained doctor can judge whether the choroidal boundaries are detected correctly based on the visual results, while in the proposed direct method, the output of CNNs is a direct value of choroidal thickness; thus, there is no other supporting information for doctors to judge whether the predicted values are reliable or not. A promising way to address this shortage is uncertainty quantification [32], which can provide a measure of trust in CNNs’ decisions. To achieve uncertainty quantification, a potential method is to combine the segmentation-based and direct methods; for instance, we can employ the segmentation-based methods to achieve an estimated value, and the direct methods to achieve another. Then, these two values would be compared: If their differences are less than a certain threshold, the estimated value would be acceptable with high confidence. Additionally, other elegant methods for uncertainty quantification would also be explored in our future research. Furthermore, the technologies, termed explainable artificial intelligence [33], that can make CNN models become more transparent could also be employed to address the limitations of the proposed method. Additionally, in a future study, we would also like to exploit a technique that can help the doctor assess the reliability of the predicted values obtained by the proposed direct method based on explainable artificial intelligence. 

In conclusion, the direct estimation of the choroidal thickness with CNNs is a very promising approach and competitive with segmentation-based methods.

## Figures and Tables

**Table 1:** The obtained results at patch and B-scan levels with different patch sizes.

	Patch Level			B−Scan Level		
Patch Size	MAE (Pixels)	ME (Pixels)	PCC	MAE (Pixels)	ME (Pixels)	PCC
200 × 50	7.1197 ± 9.1915	−1.7032 ± 11.5010	0.8644	4.5871 ± 6.4419	−1.7032 ± 7.7228	0.9193
200 × 100	8.1371 ± 10.1308	−1.3165 ± 12.9272	0.8225	5.6696 ± 7.4609	−1.3165 ± 9.2779	0.8801
200 × 200	6.0435 ± 7.5231	−1.3090 ± 9.5608	0.9041	4.3001 ± 5.8070	−1.3090 ± 7.1064	0.9274
400 × 400	5.8868 ± 7.5539	0.5584 ± 9.5607	0.8901	4.6766 ± 6.3843	0.5584 ± 7.8944	0.8969

**Table 2:** The percentage of patches in different MAE intervals.

Patch Size	MAE ≤ 5	5 < MAE ≤ 10	10 < MAE ≤ 15	MAE > 15
200 × 50	58.08%	21.59%	8.15%	12.18%
200 × 100	53.03%	22.78%	9.31%	14.88%
200 × 200	61.53%	22.40%	7.76%	8.31%
400 × 400	57.94%	28.02%	8.74%	5.29%

**Table 3:** The percentage of B-scans in different MAE intervals.

Patch Size	MAE ≤ 5	5 < MAE ≤ 10	10 < MAE ≤ 15	MAE > 15
200 × 50	74.01%	15.74%	4.45%	5.80%
200 × 100	66.06%	18.71%	6.58%	8.65%
200 × 200	75.30%	15.56%	4.83%	4.77%
400 × 400	69.68%	23.10%	4.01%	3.20%

**Table 4:** Comparison with segmentation based methods.

Methods	BM (Pixels)	CSI (Pixels)	MAE (Pixels)
Graph Cut [12]	2.4800	9.7900	-
SCA-CENet [16]	1.8640	8.5310	-
Bio-Net [17]	0.7700	4.3100	4.300
U-Net [15]	1.4500	5.8700	5.4800
Proposed	-	-	4.3001

## Data Availability

Not applicable.
